# *Anopheles hyrcanus* (Diptera: Culicidae): yet another invasive mosquito species in Germany

**DOI:** 10.1186/s13071-025-06827-7

**Published:** 2025-06-05

**Authors:** Doreen Werner, Henrike Nehls, Christiane Eska, Helge Kampen

**Affiliations:** 1 Leibniz Centre for Agricultural Landscape Research, Eberswalder Straße 84, 15374 Müncheberg, Germany; 2 Friedrich-Loeffler-Institut, Federal Research Institute for Animal Health, Südufer 10, 17493 Greifswald, Germany

**Keywords:** *Anopheles hyrcanus*, Climate change, First record, Germany, Potential vector, Spread

## Abstract

**Abstract:**

From August to October 2024, 62 specimens of the non-native mosquito species *Anopheles hyrcanus* were trapped in the federal state of Brandenburg, northeastern Germany. At one site, 59 specimens, and at a second site, 3 specimens were collected, with both sites located in floodplain areas, approximately 25 km apart. The records represent the northernmost collection sites of this species worldwide. *Anopheles hyrcanus* is considered a potential vector of malaria parasites, dirofilarial worms, and various viruses, although vector capacity appears to be generally low. The findings in Germany follow the recent detections of the species in Hungary, Slovakia, Czechia, Austria, and Poland, suggesting possible establishment in more northern areas as a consequence of climate change.

**Graphical Abstract:**

**Figure:**
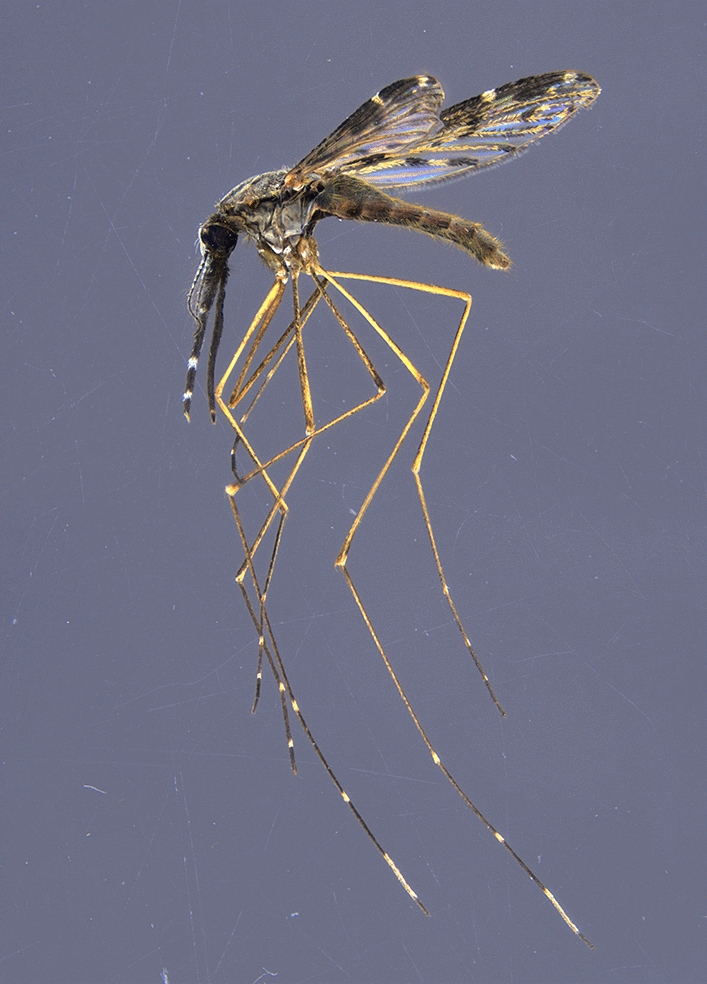

Due to globalization, and probably climate change, Germany has recently faced the invasion and establishment of several non-native mosquito species: *Aedes albopictus, Anopheles petragnani*, *Culiseta longiareolata, Aedes japonicus*, and *Aedes koreicus* [1]. The first three species are considered thermophilic and benefit from climatic change, while the *Aedes* species are of interest as potential vectors of disease agents [2]. Another non-indigenous thermophilic species, *Uranotaenia unguiculata*, which had first been detected in Germany in 1994 [3] but never been documented elsewhere in Germany, was recently found at several locations and in different years far north from its previously known single detection site in southwest Germany ([4], Werner & Kampen, unpublished data).

Except for the first report of *Ur. unguiculata*, all detections mentioned were made in the framework of a national mosquito monitoring programme launched in 2011, meant to update the occurrence, distribution, and spread of mosquitoes in Germany, with particular emphasis on vector species. The programme includes adult collections by trapping, aspirating, netting, and the citizen science project Mueckenatlas (mosquito atlas) [5], as well as larval collections [6]. In addition to the above invasive species, it looks back to the detection of several extremely rare mosquito species native to Germany but not documented for decades, such as *Ae. refiki*, *Cx. martinii*, and *An. algeriensis* [1].

In the present study, BG-Pro traps (Biogents, Regensburg, Germany) baited with CO_2_ from gas tanks (1 kg/d) were operated at several places in the Oder river region in the federal state of Brandenburg, northeastern Germany, from late May to early October 2024, but only for 10 consecutive days toward the end of the month followed by 20 or 21 days of inactivity. Traps were emptied on a daily basis, and collections were brought to the laboratory to be identified morphologically to species or complex/group level using the determination key by Becker et al. [7]. Specimens of rare species or damaged specimens were subjected to COI barcoding as described by Kampen et al. [8].

In a riparian forest along the Alte Oder river close to Quappendorf (N52.619, E14.266) and in a garden in Zeschdorf (N52.424, E14.428), *Anopheles hyrcanus* (Pallas 1771) specimens were trapped (Fig. 1). The collections also included specimens of the following culicid species/groups: *Ae. annulipes* group*, Ae. cinereus/geminus, Ae. hortensis/territans, Ae. vexans, An. claviger, An. maculipennis* complex, *An. plumbeus, Coquillettidia richiardii, Cs. annulata*, and *Cx. pipiens* complex. The bee line distance between the two sites is about 25 km. Both collection sites are located in the vast floodplain areas of the Oderbruch (River Oder lowlands), a huge natural wetland landscape of hundreds of square kilometers, and are about 12 and 10 km, respectively, away from the German–Polish border. In Quappendorf, *An. hyrcanus* specimens were captured almost daily from 23 August to 1 September 2024 to give a total of 59, whereas in Zeschdorf, 2 individuals were collected on 29 August and 1 individual was caught on 23 September 2024 (Table 1).

**Figure 1:**
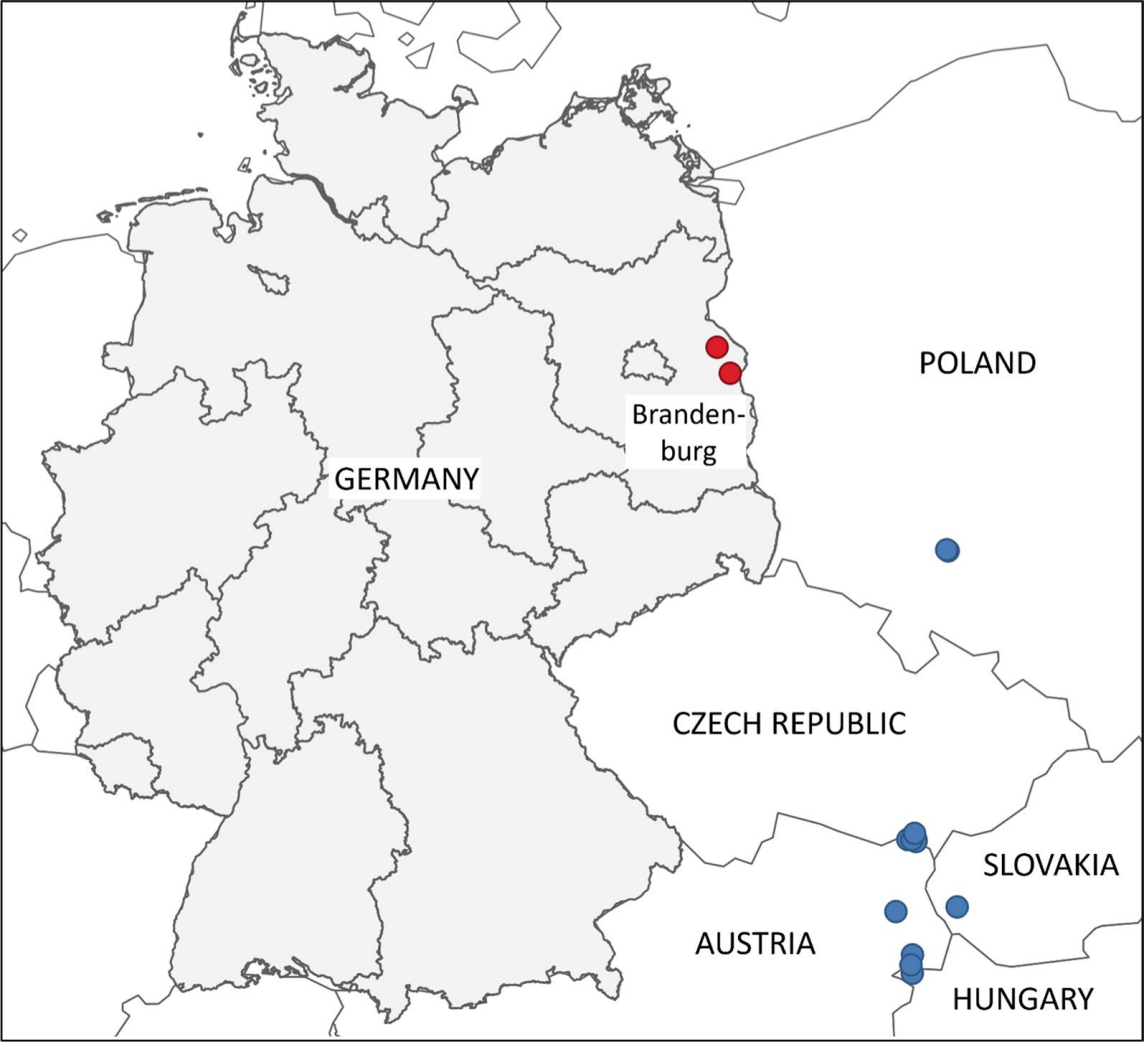
Map of Central Europe showing the most recent new country records of *Anopheles hyrcanus* in Hungary, Slovakia, Czechia, Poland (blue dots), and Germany (red dots). Collection sites according to Tóth [25], Halgoš and Benková [26], Votýpka et al. [27], Šebesta et al. [28], Lebl et al. [29], Seidel et al. [30], Lühken et al. [31], and the present study. Copyright: BKG 2023 dl-de/by-2–0: https://sgx.geodatenzentrum.de/web_public/gdz/datenquellen/Datenquellen_vg_nuts.pdf; EuroGeographics: administrative boundaries

**Table 1:** Collection of *An. hyrcanus* in the Oder river region in August and September 2024

Collection site	Collection date	Number of specimens collected per day	Total of specimens collected
Quappendorf	23 August 2024	4	59
	24 August 2024	4	
	27 August 2024	2	
	28 August 2024	2	
	29 August 2024	37	
	30 August 2024	8	
	31 August 2024	1	
	1 September 2024	1	
Zeschdorf	29 August 2024	2	3
	23 September 2024	1	

Morphological identification was done on the basis of the dichotomous key for adult *Anopheles* mosquitoes in Becker et al. [7], which leads to *An. hyrcanus* if the specimen is characterized by (i) wings possessing dark and pale scales, forming contrasting spots at least on the costa, the radius, and vein R1; (ii) two pale spots situated in the apical half of the costal margin of the wing; and (iii) the base of the fore femur being distinctly swollen. Morphological characters of the collected specimens were unambiguous for *An. hyrcanus* (Fig. 2a), with no variation between specimens being noticed. However, the tarsomeres IV of the hind legs were entirely pale scaled (Fig. 2b), suggesting *An. hyrcanus* var*. pseudopictus* on the basis of the determination key used.

**Figure 2:**
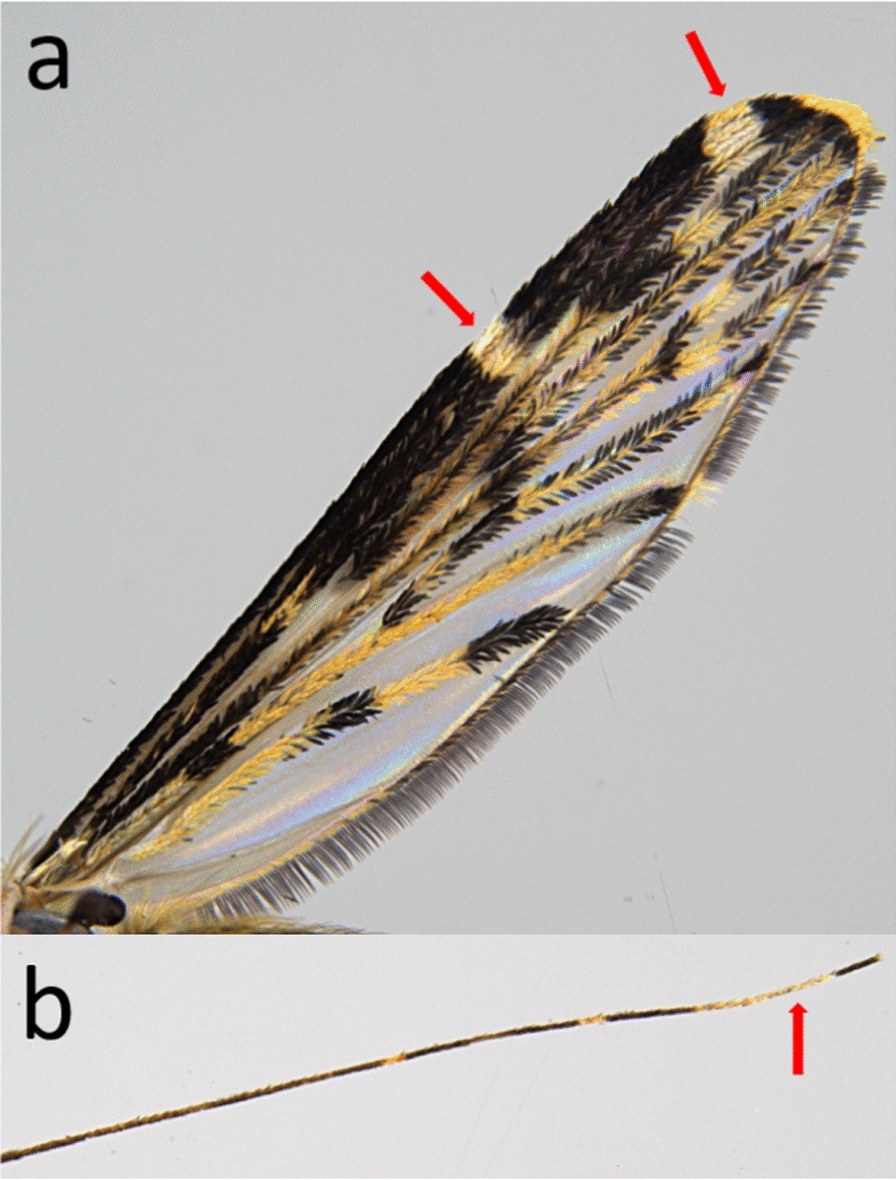
Wing and hind leg of specimens collected in the present study. According to Becker et al. [10], the two pale spots in the apical half of the costal margin of the wing (**a**) is indicative for *An. hyrcanus*, while the entirely pale-scaled tarsomere IV of the hind leg (**b**) suggests *An. hyrcanus* var. *pseudopictus*

For confirmation, one specimen of each of the two collection sites was processed by COI barcoding. The COI DNA sequences were 99.86% identical to each other (with one single bp difference out of 710 nucleotides), and 100% and 99.85%, respectively, identical to the best matching sequence entries in the Barcode of Life database (BOLD: https://boldsystems.org), which represented *An. hyrcanus* and *An. pseudopictus*. The COI regions of these two taxa are highly similar, causing both Ponçon et al. [9] and Djadid et al. [10] to suggest that they are one and the same species. Even previously, a great variability in the extent of the pale ringing of the hind legs of *An. hyrcanus* was reported [11], and some authors had speculated *An. pseudopictus* to be just a different-colored variant of *An. hyrcanus* [12–15]. Accordingly, Becker et al. [7] do not treat *An. pseudopictus* as a distinct species but as a variety of *An. hyrcanus*, although officially, *An. pseudopictus* is still considered a true species [16]. The COI sequences produced in this study have been deposited in GenBank under accession numbers PV039093 and PV039678.

*Anopheles hyrcanus* belongs to a group of about 30 closely related species (Hyrcanus Group), three of which (according to valid systematics [16], although by some authors considered synonymous (e.g., [17, 18])) occur in the western Palaearctic: *An. hyrcanus* (Pallas 1771), *An. pseudopictus* Grassi 1899, and *An. chodukini* Martini 1929 [19, 20]. According to Becker et al. [7], species of the Hyrcanus Group develop in open water bodies in floodplain areas, marshes, and irrigated rice fields where they can reach particularly high abundances (e.g., [21, 22]). Until recently, they were known to occur from the Iberian Peninsula, through Europe south of the Alps, and Asia south of about 50 °N to the Pacific [19, 23], indicating adaptation to warm climates. However, first reports from more northern countries such as Hungary (1995–2003; exact year of detection not provided), Slovakia (2002 and 2003), Czechia (2005–2007), Austria (2012), and Poland (2019) have recently been published [24–30] (Fig. 1). On the basis of these, the findings in Germany suggest a northward spread which is probably facilitated by climate change [25]. For Serbia, temperature increase as a prerequisite condition for the spread of *An. hyrcanus* has impressively been illustrated by Mihailović et al. [31].

A total of 17 specimens of the collected *An. hyrcanus* were blood-fed, although poorly engorged. To determine vertebrate host species, blood meal analysis was carried out following Köchling et al. [32]. Identification of the blood origin was successful in 11 cases: 6 females had fed on humans, 2 females each on boar and wolf, and 1 female on sheep. *Anopheles hyrcanus* is believed to feed preferentially on mammals, and feeding on humans and sheep as in this study has been described repeatedly [19, 33]. Occasionally, it has also been found having fed on birds [34], in agreement with the detection of specimens infected with avian plasmodia in Austria [29]. The uncommon and previously not described finding of wolf as a blood host of specimens collected in the present study might be explainable by the recent spread of wolves in Germany, which have become particularly abundant in the federal state of Brandenburg [35]. It may indicate a generalist biting behavior of *An. hyrcanus*.

Seasonal activity of adult *An. hyrcanus* has been reported to occur from May to September, with a peak from mid-June to mid-September [7, 22]. The observations by Votypka et al. [26] (late June to August), Lebl et al. [28] (May to September), and Lühken et al. [30] (July to September) are in line with the collection period of this study.

The fact that 62 specimens were collected at two places 25 km apart strongly argues against isolated random findings caused by the collection strategy that was not targeted at *An. hyrcanus*. Rather, it can be assumed that *An. hyrcanus* has become established in Germany. Establishment provided, the detection increases the German mosquito fauna to 53 species, including 9 of the genus *Anopheles* [1, 36]. It also adds another potential vector species. In contrast to numerous articles in which *An. hyrcanus* is presented as a demonstrated vector, no immediate evidence of pathogen transmission by this species exists, and pertinent laboratory infection studies are missing. Most of these uncertainties must be attributed to systematic revision and changing taxonomy since most species of the present Hyrcanus Group were once considered “races” of *An. hyrcanus* before elevating them to species level [16, 19, 37, 38]. If races are not mentioned in the historic literature, the present species status might be deduced, but with some uncertainty, only from the geographic origin of the formerly investigated specimens. Thus, decade-old studies on pathogen infection and vector competence of *An. hyrcanus* carried out in the eastern Palaearctic and the Oriental regions (e.g., [13, 39]) probably refer to present-day *An. sinensis, An. nigerrimus, An. lesteri*, and other species of the Hyrcanus Group distributed there. Notwithstanding, there are strong indications for *An. hyrcanus* to be a vector of malaria parasites: field-collected specimens have been shown to be infected with malarial parasites in the salivary glands [38, 40]. Together with regionally high abundances and high anthropophily, this makes it a likely vector of plasmodia [22, 33, 41–43], although probably a secondary one only due to high degrees of exophagy and exophily [33]. Furthermore, *Plasmodium falciparum, Dirofolaria immitis*, and *D. repens*, as well as Japanese encephalitis, West Nile, Sindbis, and Ťahyňa viruses have been isolated from, or genetically detected in, total homogenized *An. hyrcanus* specimens [10, 44–50].

The finding of still another invasive mosquito species in Germany demonstrates the importance of mosquito surveillance. Considering continuing globalization and global warming, it must be expected that *An. hyrcanus* will not be the last thermophilic potential mosquito vector species emerging and establishing in Central Europe.

## Data Availability

Data are provided within the manuscript or supplementary information files.
